# Trained Lifeguards Performing Pediatric Cardiopulmonary Resuscitation While Running: A Pilot Simulation Study

**DOI:** 10.3390/children10081348

**Published:** 2023-08-04

**Authors:** Myriam Santos-Folgar, Antonio Rodriguez-Nunez, Roberto Barcala-Furelos, Martín Otero-Agra, Santiago Martínez-Isasi, Felipe Fernández-Méndez

**Affiliations:** 1 REMOSS Research Group, Faculty of Education and Sport Sciences, Universidade de Vigo, 36005 Pontevedra, Spain; m.santos.folgar@gmail.com (M.S.-F.); roberto.barcala.furelos@gmail.com (R.B.-F.); felipefernandez@uvigo.es (F.F.-M.); 2 School of Nursing, Universidade de Vigo, 36004 Pontevedra, Spain; 3 Department of Obstetrics, Complexo Hospitalario of Pontevedra, 36001 Pontevedra, Spain; 4 CLINURSID Research Group, Psychiatry, Radiology, Public Health, Nursing and Medicine Department, Universidade de Santiago de Compostela, 15706 A Coruña, Spain; smtzisasi@gmail.com; 5 Simulation and Intensive Care Unit of Santiago (SICRUS) Research Group, Health Research Institute of Santiago, University Hospital of Santiago de Compostela (CHUS), 15706 A Coruña, Spain; 6 Faculty of Nursing, Universidade de Santiago de Compostela, 15782 A Coruña, Spain; 7 Paediatric Critical, Intermediate and Palliative Care Section, Hospital Clínico Universitario de Santiago de Compostela, 15706 A Coruña, Spain; 8 Collaborative Research Network Orientated to Health Results (RICORS): Primary Care Interventions to Prevent Maternal and Child Chronic Diseases of Perinatal and Developmental Origin, RD21/0012/0025, Instituto de Salud Carlos III, 28029 Madrid, Spain

**Keywords:** infant, cardiopulmonary resuscitation (CPR), lifeguards, walking, running

## Abstract

The aim of this study was to compare the quality of standard infant CPR with CPR in motion (i.e., walking and running) via performing maneuvers and evacuating the infant from a beach. Thirteen trained lifeguards participated in a randomized crossover study. Each rescuer individually performed three tests of 2 min each. Five rescue breaths and cycles of 30 chest compressions followed by two breaths were performed. Mouth-to-mouth-and-nose ventilation was carried out, and chest compressions were performed using the two-fingers technique. The manikin was carried on the rescuer’s forearm with the head in the distal position. The analysis variables included compression, ventilation, and CPR quality variables, as well as physiological and effort parameters. Significantly lower compression quality values were obtained in running CPR versus standard CPR (53% ± 14% versus 63% ± 15%; *p* = 0.045). No significant differences were observed in ventilation or CPR quality. In conclusion, lifeguards in good physical condition can perform simulated infant CPR of a similar quality to that of CPR carried out on a victim who is lying down in a fixed position.

## 1. Introduction

In the event of a pediatric cardiac arrest taking place in a dangerous situation or at a remote location, it is essential to evacuate the victim as soon as possible. However, it is still critical to begin resuscitation maneuvers early in order to improve the chances of survival [1,2,3,4]. Therefore, a major dilemma may arise: do we simply start resuscitation maneuvers early, thus delaying evacuation? Or do we evacuate the pediatric victim immediately and postpone early initiation of the resuscitation maneuvers? The fundamental issue is that on-scene resuscitation is not always feasible (due to the conditions of the location or the hazards of the environment), so the dilemma becomes whether to evacuate quickly or to evacuate while attempting some measure of cardiac arrest mitigation.

Since the 1990s, the American Heart Association (AHA) has advised that it is possible to carry a pediatric victim (infant) in arms while the steps of cardiopulmonary resuscitation (CPR) are initiated [5]. A study by our group showed that CPR quality can be maintained in a simulated walking CPR scenario [6]. However, there are still gaps in the knowledge relating to this issue.

To minimize response time, a pediatric victim could be carried in the rescuer’s arms; then, resuscitation maneuvers would be performed while running and evacuating the victim. However, we do not know if the time gained via running would be worthwhile if the CPR quality deteriorated greatly. To our knowledge, no study published to date has researched this topic. We hypothesized that lifeguards in good physical condition who were trained in basic life support would be able to perform quality CPR. Therefore, this research was carried out with the aim of comparing the quality of standard CPR (S-CPR) versus walking CPR (W-CPR) and running CPR (R-CPR) in a simulated scenario on a beach.

## 2. Materials and Methods

### 2.1. Study Design

**Figure 1:**
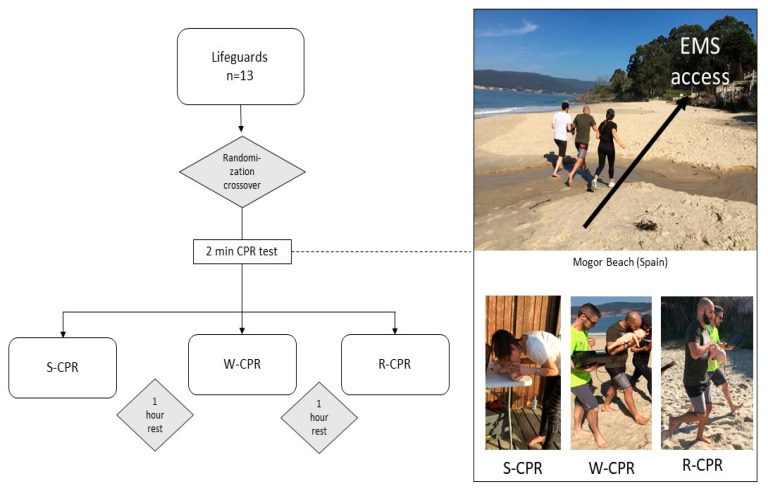
Flowchart.

A randomized crossover simulation study was used to compare standard CPR (S-CPR) with an infant manikin on a firm surface versus walking CPR (W-CPR) and running CPR (R-CPR) with an infant manikin on the rescuer’s forearm while performing the maneuvers of resuscitation (chest compressions and mouth-to-mouth-and-nose ventilation) (Figure 1).

### 2.2. Study Participants

Thirteen lifeguards were asked to perform 2 min of CPR on an infant manikin in three different scenarios. The young and healthy rescuers were in a good physical condition suitable for water rescue; lifeguards undergo physical tests every year for the renewal of their contract, and all participants had a valid professional license at the time of the research. In addition, the lifeguards made a subjective assessment of their physical condition on a 0–10 scale.

Before the investigation, all participants had attended a one-hour training course in basic pediatric life support. Participants trained individually, using standard CPR on an infant simulator while obtaining feedback, until they were able to perform quality CPR (over 70%) [7]. The training was conducted by two instructors who specialized in pediatric CPR. Prior to data collection, all participants were familiarized with the manikin on which the tests were to be performed.

All participants were informed about the study and gave their written informed consent. The research respected the ethical principles of the Declaration of Helsinki.

### 2.3. Study Protocol

Each participant randomly performed 2 min of CPR in three situations: (A) S-CPR, (B) W-CPR, and (C) R-CPR. During the S-CPR test the manikin was on a firm surface. In the W-CPR and R-CPR tests the infant manikin was carried on the rescuer’s forearm with the head in the distal position. Rescuers did not receive any feedback during the tests.

The order of the three tests performed by each participant was randomized to minimize possible biases related to previous experience. A randomization by blocks was performed, so that the following order blocks were created:

(1) S-CPR/W-CPR/R-CPR
(2) S-CPR/R-CPR/W-CPR
(3) W-CPR/S-CPR/R-CPR
(4) W-CPR/R-CPR/S-CPR
(5) R-CPR/S-CPR/W-CPR
(6) R-CPR/W-CPR/S-CPR

After this block selection, each participant was randomly assigned to one of the blocks. Research Randomizer (www.randomizer.org, accessed on 21 February 2020) computer software was used to assign the order in which the tests would be performed.

The recommendations of the European Resuscitation Council (ERC) 2021 [8] for infants were followed: the participants performed CPR individually, starting with 5 rescue breaths followed by cycles of 30 chest compressions and 2 breaths [9]. The compressions were performed using the two-fingers technique while the ventilations were carried out via the mouth-to-mouth-and-nose method.

A minimum rest period of 1 h was set between each test (Figure 1). After each CPR test, the participants made a subjective assessment of their perceived fatigue using Borg’s RPE scale [10].

### 2.4. Conditions

Data capture was carried out on Mogor Beach in Marín (Northwest Spain), beginning at the following GPS location: 42°23′09.4″ N–8°43′09.8″ W.

### 2.5. Variables and Measuring Equipment

#### 2.5.1. Cardiopulmonary Resuscitation Variables

Two groups of variables were evaluated.

- Chest Compressions (CC): Number of CC; Mean depth in mm; Mean rate in CC/min; Percentage of CC with adequate depth; Percentage of CC with adequate rate; Percentage of CC with adequate release; Percentage of CC with adequate hand position.
- Ventilations (V): Number of total V; Mean pause time for V in s; Number of effective V; Number of V with adequate volume; Number of V with excessive volume; Number of V with insufficient volume; Mean volume in ml.

For data analysis, a Laerdal Resusci Baby QCPR Wireless SkillReporter^®^ (Stavanger, Norway) manikin was used, with Laerdal Resusci Anne Wireless software (Stavanger, Norway) version 2.0.0.14. The configuration parameters were: tidal volume 6–10 mL/kg (35–55 mL) [11,12,13], CC depth 36–44 mm, and CC rate 100–120 CC/min, in accordance with the 2021 ERC guidelines [8]. The manufacturer does not specify the age of the Resusci Baby QCPR, so the growth charts of the World Health Organization (WHO) [14] were used to identify its anthropometric parameters. The manikin corresponds to a 3-month-old baby.

#### 2.5.2. CPR Quality Variables

Quality parameters were evaluated and disaggregated in CC quality, V quality, and CPR quality. Each variable was expressed as a percentage; variable calculations were based on the following formulas published in previous studies [6,15]:

- CC quality, calculated using the formula: (CC with adequate depth + CC with correct chest recoil + CC with adequate rate) ÷ 3.
- V quality, calculated using the formula: Number of V with adequate volume ÷ Number of total V × 100.
- CPR quality, calculated using the formula: [(CC quality + V quality) ÷ 2].

#### 2.5.3. Physiological and Effort Parameters

For the physiological analysis, the percentage of maximum Heart Rate during CPR (%HRmax) was measured. The HR was measured with a Polar Team sensor (Kempele, Finland). The Rating of Perceived Exertion (RPE) was measured on a 0–10 scale [10]. RPE measurement using a modified Borg’s scale is common for estimating physical effort in CPR studies [6,15,16,17].

### 2.6. Statistical Analysis

The sample size was based on an assumed minimum effect size of 0.50, an alpha error probability of 0.05, and a statistical power of 0.95. These assumptions required a sample size of 12 study participants as computed by G*Power 3.1.9.2 software (Heinrich Heine University, Düsseldorf, Germany). A final sample size of 13 participants was selected to perform this study.

All statistical analyses were performed with SPSS version 20 for Windows (SPSS Inc., IBM, Chicago, IL, USA). The variables were described using measures of central tendency (median and mean), dispersion [interquartile range (IQR) and standard deviation (SD)], or 95% confidence intervals. For comparisons, the Shapiro–Wilk test was used first to check the normality of the data. For parametric variables, a repeated measures ANOVA test with Bonferroni correction was used, and for non-parametric variables comparisons were analyzed using the Friedman test with the Bonferroni correction and the Wilcoxon signed- rank test. For effect size, both Cohen’s d test and Rosenthal’s r test were used. A significance value of 0.05 was set for all tests.

## 3. Results

A total number of 39 tests were performed by 13 lifeguards (85% were men). The median age of the participants was 26 years (IQR: 23–29 years), the median height was 173 cm (IQR: 166–180 cm), and the median weight was 81 kg (IQR: 64–88 kg).

### 3.1. Cardiopulmonary Resuscitation Variables (Table 1)

In the CC analysis, significantly lower results were found in CC with adequate release when comparing R-CPR (63%; IQR: 27–84%) to S-CPR (95%; IQR: 55–98%; *p* = 0.032; ES = 0.50) and W-CPR (89%; IQR: 67–97%; *p* = 0.018; ES = 0.54). Lower values were also seen in CC with adequate hand position in R-CPR (85%; IQR: 63–91%) when compared to S-CPR (100%; IQR: 93–100%; *p* = 0.005; ES = 0.62. No differences were observed in the rest of the CC variables. In the V analysis, significant differences were found in Mean pause time for V, in which the R-CPR test presented significantly lower values (4 s; IQR: 4–5 s) than the S-CPR test (5 s; IQR: 5–7 s; *p* = 0.024; ES = 0.52). The mean volume was significantly higher in the S-CPR test (53 mL; IQR: 45–63 mL) than in the W-CPR (44 mL; IQR: 38–58 mL; *p* = 0.010; ES = 0.58) and R-CPR (44 mL; IQR: 41–60 mL; *p* = 0.010; ES = 0.58) tests. Differences were observed in the rest of the V variables.

### 3.2. Quality CPR Variables (Figure 2)

In relation to the Quality of CC, significantly lower values were observed in R-CPR (53 ± 14%; 95% CI: 45–62%) than in S-CPR (63 ± 15%; 95% CI: 64–72%; *p* = 0.045; ES = 0.71). The W-CPR presented a CC Quality of 60 ± 16%; 95% CI: 50–69%. Regarding the Quality of V, no significant differences were observed between the three tests; the medians of quality in values ranged between 43% and 48%. Nor were any differences observed in the Quality of CPR, the best value being obtained in S-CPR (56 ± 20%; 95% CI: 43–68%) followed by W-CPR (52 ± 12%; 95% CI: 44–59%) and R-CPR (48 ± 13%; 95% CI: 41–56%).

### 3.3. Physiological and Effort Parameters (Figure 3)

Physical condition had a median of 7.0 out of 10.0 (IQR: 4.5–7.5). During the 2 min of R-CPR, rescuers covered 374 m (IQR: 358–392 m), significantly more than during W-CPR (201 m; IQR: 196–258 m; *p* = 0.002; ES = 0.61). This represents an average speed of 11.2 km/h (IQR: 10.7–11.8 km/h) during R-CPR and 6.0 km/h (IQR: 5.8–7.7 km/h) during W-CPR (*p* = 0.002). For this, the participants needed to take 334 steps (IQR: 333–357 steps) during R-CPR and 270 steps (IQR: 267–293 steps) during W-CPR (*p* = 0.002). R-CPR represented a significant physiological increase over W-CPR and S-CPR. Similarly, W-CPR also represented a significant increase over S-CPR [S-CPR: 65 ± 12% (95% CI: 57–73%); W-CPR: 89 ± 8% (95% CI: 84–94%); R-CPR: 98 ± 6% (95% CI: 94–102%)]. The same occurred when assessing RPE [S-CPR: 2 (IQR: 1–3) W-CPR: 6 (IQR: 5–7); R-CPR: 9 (IQR: 8–10)].

## 4. Discussion

CPR recommendations for risky situations or isolated environments are ambiguous. Consequently, rescuers may not know what to do when resuscitation maneuvers must be started early while simultaneously evacuating the victim to remove them from danger or obtain specialized healthcare support. Since it is not feasible to carry out clinical trials with patients to resolve these doubts, simulated and controlled scenarios can give us an idea of what could happen with real victims.

In a previous study by our group that used a similar methodology, we demonstrated that good CPR can be performed while walking, although we were left wondering whether the distance and time spent would be appropriate in certain real-life circumstances. For this reason, we have carried out the current study, in which the participants had to perform running CPR in order to reach the point of “advanced” or “safe” care as soon as possible.

Our results indicate that: (a) R-CPR is possible, although the total quality presents a slight, non-significant decrease, (b) the quality of compressions during R-CPR decreases by 10% compared to S-CPR, but there are no differences with respect to W-CPR, (c) no differences were observed in airway management during the CPR tests.

This research provides novel data on R-CPR. To date, no studies have been published that address this issue. The overall quality of the CPR was very similar in all three simulations. Furthermore, the only published study that evaluated W-CPR did not observe significant differences in total quality either, with values very similar to those of our study (S-CPR: 59% and W-CPR: 49%) [6]. In addition, the quality results of our investigation were similar to those reported by other investigations using standard infant CPR [13].

High-quality chest compression is very important for the survival of patients in cardiac arrest [18]. Our results show that the quality of compressions decreases by 10% during R-CPR compared to standard CPR. This is mainly due to a failure in chest release and hand position. However, these two parameters can be improved. It is logical that the rescuer’s hands move when running, making it difficult to maintain the cardiac massage point and the correct chest release. Therefore, it is essential to undergo CPR training in these conditions (walking and running) in order to improve compression skills during movement. However, it is not usual to train in these conditions since the recommendations are not clear.

The trained rescuers in our research obtained compression quality values of around 50%. Although recent research shows that professional lifeguards can perform compressions with over 80% quality on an infant manikin [15], Martin et al. reported that even trained rescuers have difficulty performing adequate compressions during simulated infant CPR [19].

A systematic review and meta-analysis found that the rate of chest compressions is associated with survival outcomes [20]. Therefore, performing compressions with an adequate rhythm is essential. Although our results did not show significant differences in the rate of compressions, the rate was above the recommended rate in all three tests, and even well-trained rescuers in a standing position maintained a rate of compressions above the recommended rate.

Studies in adults and animals have observed that incomplete chest recoil negatively affects hemodynamics, since it increases intrathoracic pressure and decreases venous return to the heart [21,22,23,24,25]. It also decreases coronary and cerebral pressures, and this effect continues for a significant period of time after the cessation of incomplete chest release [24]. Therefore, even short periods of incomplete chest recoil during CPR can have harmful effects on tissue perfusion and CPR outcomes [26]. During standard CPR almost 100% of compressions were performed with correct chest recoil. However, during CPR in motion this percentage decreased to almost 60%. This may be related to the rate of compressions, since a rate of compressions greater than 110/minute was associated with a very low percentage of complete recoil of the thorax (<18%) [27]. Another possible cause may be that the speed of the rescuer makes complete chest release difficult.

Donoghue et al. showed that the depth of compressions is a deficient skill in the rescue of infant victims [28]. An unexpected result of our study was that rescuers had no difficulty performing compressions of adequate depth with the manikin on their forearm, despite it not being on a firm surface. This may be related to the good physical condition of the rescuers, since it has been reported that physical training has a positive impact on chest compressions [29]. However, we must take into account that the evaluation of physical condition was based on the subjective perception of the rescuers.

Similar to our study, Smereka et al. observed that the optimal way to perform infant resuscitation was on the rescuer’s forearm in a static position, although this research focused on newborn CPR simulations [30]. However, the results of our study contrast with a recently published study on W-CPR that observed poor compression depth during brisk walking CPR [6]. In the same way, Mühlbacher et al. showed a decrease in the depth of compressions during simulated CPR with the infant on the forearm; however, they did not perform CPR in motion [31].

A surprising and positive result of the current study was that rescuers did not have difficulty performing rescue breaths during CPR on the move, as was the case in the W-CPR investigation performed by trained lay rescuers [6]. However, it has been observed that during CPR administered to adult manikins in rescue watercraft, the quality of mouth-to-mouth ventilations worsened dramatically as craft speed increased [32]. Our results suggest that transporting the infant manikin with the head in a distal position, while supporting it with the hand, seems to be beneficial and may favor opening the airway with a simple movement of the wrist.

Fatigue negatively affects the quality of an activity which requires significant physical effort, such as CPR [33,34]. In our current study we used Borg’s rating of perceived exertion (RPE) scale to assess the subjective fatigue perceived by rescuers. As expected, during R-CPR perceived fatigue was much higher than during S-CPR or W-CPR. During R-CPR, fatigue reached critical values (9 out of 10); R-CPR is considered a very hard activity according to a modified Borg’s scale (0–10) [10]. Even so, CPR was not affected, possibly because the effort time was relatively short. These fatigue results coincide with the fatigue reported by nursing students who performed CPR on infant manikins for a prolonged period of time. However, that study measured fatigue using the Visual Analogic Scale (VAS) [35]. Similarly, other investigations reported severe fatigue during infant CPR using the two-finger technique [36,37].

This study has several implications for practice and is based on the research team’s experience in a real pediatric emergency situation that gave rise to the research question. In the education and training of first responders, priority should be given to the most common actions related to prevention, but special resuscitation situations should also be considered. Although these are rare, they require specialized preparation and training. Trained lifeguards can be an asset during a pediatric cardiac arrest. According to the results of this research, trained rescuers can perform acceptable pediatric walking and running CPR even under highly fatiguing conditions.

The results of this research are not limited to beaches that are difficult to access but can apply to any aquatic areas (lakes, rivers, marshes, or conventional swimming pools) where there is no road access for an ambulance or where there are difficulties in accessing specialized help.

This research has several limitations that should be mentioned. On the one hand, it was carried out in a simulated environment, so motivational factors and stress are different from real life. On the other hand, varying distances, weather conditions, locations, or types of rescuers, respectively, could result in different CPR outcomes. In addition, alternative CPR times could provide different results; 2-min tests were performed in the current study, and further research is needed on longer duration CPR while walking or running. As with all simulation studies, the results cannot be directly extrapolated to the care provided to real victims. The strengths of this study are the novelty and relevance of the topic, as well as the limited previous evidence in this context.

## 5. Conclusions

Under simulated CPR conditions, as rescuers transport an infant victim to safety or to seek expert help while walking or running, lifeguards in good physical condition can perform CPR of a similar quality to when the victim is lying down in a fixed position on a hard surface. The components of CPR quality that worsen under moving conditions are chest compression release and hand position.

CPR courses should include practice with victim evacuation scenarios. Moreover, during such trainings instructors should reinforce the following aspects of CPR: frequency, chest compression release, and hand position.

## Figures and Tables

**Figure 2:**
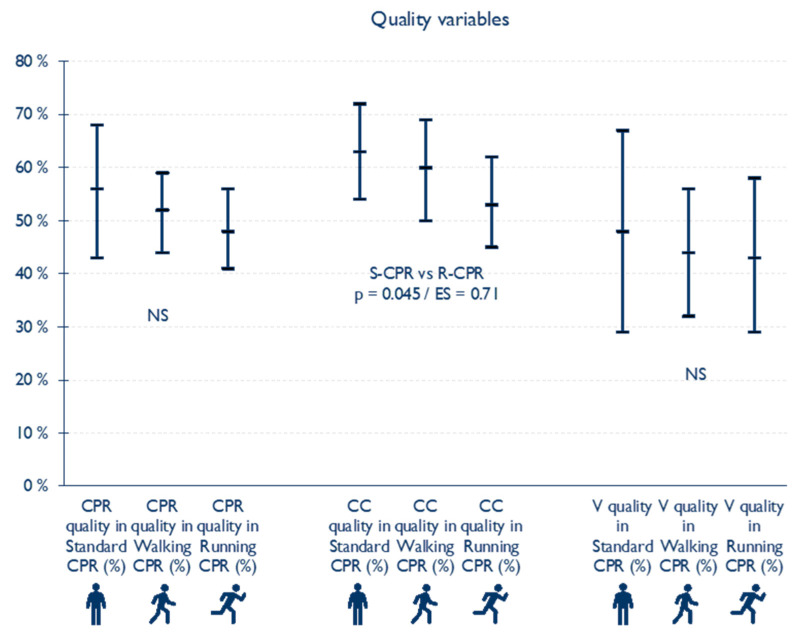
CPR, CC and V quality variables.

**Figure 3:**
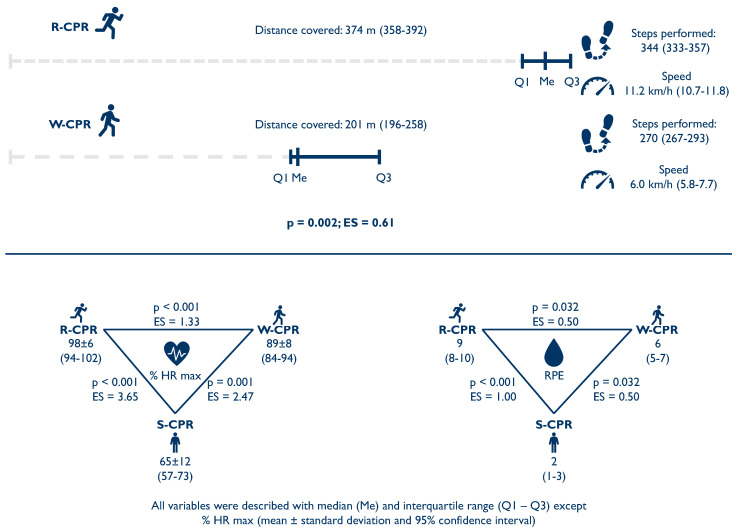
Physiological and effort parameters.

**Table 1:** Quality of chest compressions and ventilations while standing, walking, or running (N = 13).

Variables	Standard CPR (S-CPR)		Walking CPR (W-CPR)		Running CPR (R-CPR)		Significance
	Me	IQR	Me	IQR	Me	IQR	
**CC: Chest compressions**							
**Number of CC**	180	(169–190)	199	(170–212)	191	(174–243)	NS ^†^
**Mean depth (mm)**	42	(41–43)	41	(37–43)	42	(41–43)	NS ^†^
**Mean rate (CC/min)**	129	(121–142)	130	(119–154)	133	(115–176)	NS ^†^
**CC with adequate depth (%)**	99	(98–100)	96	(64–99)	95	(77–96)	NS ^†^
**CC with adequate rate (%)**	1	(0–46)	8	(2–29)	7	(1–37)	NS ^†^
**CC with adequate release (%)**	95	(55–98)	89	(67–97)	63	(27–84)	S vs. R = 0.032 (0.50) ^†^ W vs. R = 0.018 (0.54) ^†^
**CC with adequate hand-position (%)**	100	(93–100)	92	(68–99)	85	(63–91)	S vs. R = 0.005 (0.62) ^†^
**V: Ventilations**							
**Mean pause time for V (s)**	5	(5–7)	4	(4–5)	4	(4–5)	S vs. R = 0.024 (0.52) ^†^
**Number of effective V**	15	(15–16)	16	(13–17)	15	(13–19)	NS ^†^
**Number of V with adequate volume**	7	(4–10)	7	(5–10)	7	(3–13)	NS *
**Number of V with excessive volume**	6	(0–10)	3	(1–7)	3	(1–7)	NS ^†^
**Number of V with insufficient volume**	0	(0–4)	5	(1–7)	5	(4–7)	NS ^†^
**Mean volume (mL)**	53	(45–63)	44	(38–58)	44	(41–60)	S vs. R = 0.010 (0.58) ^†^ S vs. W = 0.010 (0.58) ^†^

Me: Median. IQR: Interquartile range (Q1–Q3). * Repeated measures Student’s *t* test (*p* = 0.05). In brackets: Effect Size (Cohen’s d test). ^†^ Wilcoxon signed-rank test (*p* = 0.05). In brackets: Effect Size (Rosenthal’s r test). Effect Size: (<0.2: Trivial/0.2–0.5: Small/0.5–0.8: Moderate/0.8–1.3: Large/>1.3: Very large).

## Data Availability

Not applicable.
